# Enhanced Interplay of Neuronal Coherence and Coupling in the Dying Human Brain

**DOI:** 10.3389/fnagi.2022.813531

**Published:** 2022-02-22

**Authors:** Raul Vicente, Michael Rizzuto, Can Sarica, Kazuaki Yamamoto, Mohammed Sadr, Tarun Khajuria, Mostafa Fatehi, Farzad Moien-Afshari, Charles S. Haw, Rodolfo R. Llinas, Andres M. Lozano, Joseph S. Neimat, Ajmal Zemmar

**Affiliations:** ^1^Department of Neurosurgery, Henan Provincial People’s Hospital, Henan University People’s Hospital, Henan University School of Medicine, Zhengzhou, China; ^2^Institute of Computer Science, University of Tartu, Tartu, Estonia; ^3^Division of Neurosurgery, Department of Surgery, Vancouver General Hospital, University of British Columbia, Vancouver, BC, Canada; ^4^Division of Neurosurgery, Department of Surgery, Toronto Western Hospital, University of Toronto, Toronto, ON, Canada; ^5^Epilepsy Program, Faculty of Medicine, University of British Columbia, Vancouver, BC, Canada; ^6^Department of Neuroscience and Physiology, New York University Grossman School of Medicine, New York, NY, United States; ^7^Department of Neurosurgery, School of Medicine, University of Louisville, Louisville, KY, United States

**Keywords:** coherence, coupling, near-death, end-of-life, life recall

## Abstract

The neurophysiological footprint of brain activity after cardiac arrest and during near-death experience (NDE) is not well understood. Although a hypoactive state of brain activity has been assumed, experimental animal studies have shown increased activity after cardiac arrest, particularly in the gamma-band, resulting from hypercapnia prior to and cessation of cerebral blood flow after cardiac arrest. No study has yet investigated this matter in humans. Here, we present continuous electroencephalography (EEG) recording from a dying human brain, obtained from an 87-year-old patient undergoing cardiac arrest after traumatic subdural hematoma. An increase of absolute power in gamma activity in the narrow and broad bands and a decrease in theta power is seen after suppression of bilateral hemispheric responses. After cardiac arrest, delta, beta, alpha and gamma power were decreased but a higher percentage of relative gamma power was observed when compared to the interictal interval. Cross-frequency coupling revealed modulation of left-hemispheric gamma activity by alpha and theta rhythms across all windows, even after cessation of cerebral blood flow. The strongest coupling is observed for narrow- and broad-band gamma activity by the alpha waves during left-sided suppression and after cardiac arrest. Albeit the influence of neuronal injury and swelling, our data provide the first evidence from the dying human brain in a non-experimental, real-life acute care clinical setting and advocate that the human brain may possess the capability to generate coordinated activity during the near-death period.

## Introduction

Near-death experience (NDE) has been reported in situations where the brain transitions toward death. Subjective descriptions of this phenomenon are described as intense and surreal and include a panoramic life review with memory recalls, transcendental and out-of-body experiences with dreaming, hallucinations and a meditative state (Vanhaudenhuyse et al., 2007). The neurophysiological signature of this phenomenon is unclear. It is hypothesized that the brain may generate a memory replay within this “unconscious” phase with an increase in oscillatory activity (Mobbs and Watt, 2011; Facco and Agrillo, 2012; Greyson et al., 2012; Borjigin et al., 2013). In healthy subjects, neural oscillations provide a temporal frame for information processing of perception, consciousness and memory during waking, dreaming and meditation (Llinás and Paré, 1991; Llinas and Ribary, 1993; Llinás et al., 1998; Lutz et al., 2004; Beauregard and Paquette, 2008; Fries, 2009). Particularly, enhanced thalamocortical activity, increase of gamma power and long-range gamma synchronization (> 35Hz) has been identified in conscious perception (Llinás et al., 1998; Tononi et al., 1998; Rodriguez et al., 1999; Singer, 2001; Varela et al., 2001). Alpha-band oscillations are the dominant band in the human brain, important for information processing, especially in the visual cortex, and are likely to have an inhibitory function on cortical areas that are not in use (Klimesch, 2012). A similar inhibitory function also has been suggested for delta band activity, which may suppress networks that are not essential for task accomplishment (Harmony, 2013). Theta rhythms play a critical role in memory recall, especially in verbal and spatial memory tasks as well as during meditation (Kahana et al., 2001; Siapas et al., 2005). The intricate interplay among these bands and cross-frequency coupling account for long-range neuronal communication, perception and memory retrieval (Canolty et al., 2006; Jensen and Colgin, 2007; Tort et al., 2009; Harmony, 2013). As such, memory flashbacks during recall of NDEs have been linked with oscillatory activity, similar to real life memory recall (Chawla et al., 2009, 2017; Palmieri et al., 2014).

The classic view of a hypoactive brain during the near-death phase has been challenged by recent evidence demonstrating end-of-life electrical surges (ELES) (Chawla et al., 2009, 2017; Mays and Mays, 2011). In rodents, increased cortico-cardiac and anterior-posterior connectivity, phase-coupling between gamma oscillations to alpha and theta waves and an increase in gamma-band oscillatory activity was identified in the first 30 s after cardiac arrest (Borjigin et al., 2013; Li et al., 2015). Beside cardiac arrest, a surge of gamma oscillations has been observed immediately upon asphyxia and hypercapnia (Li et al., 2015; Martial et al., 2020). Thus far, reports investigating the neural correlates of NDEs stem from experimental studies in animals, from measurements that were obtained during NDE recall, rather than real-time recording during the NDE itself, or from simplified EEG recordings in palliative patients (Borjigin et al., 2013; Palmieri et al., 2014; Martial et al., 2020). The neurophysiological processes occurring in the dying human brain have yet, to our understanding, not been reported for patients in real-life acute settings since capturing of full standard EEG activity in the transitory phase to death is rare and cannot be planned experimentally. Here, we report what is to our knowledge the first continuous EEG recording from the human brain in the transition phase to death. We find decreased theta activity and an increase of absolute gamma power after bilateral suppression of neuronal activity. Post cardiac arrest, relative gamma-band power is increased while delta, beta and alpha bands show reduced activity. Finally, we observe strong modulation of narrow- and broad-band gamma activity by the alpha band.

## Case Report

**FIGURE 1:**
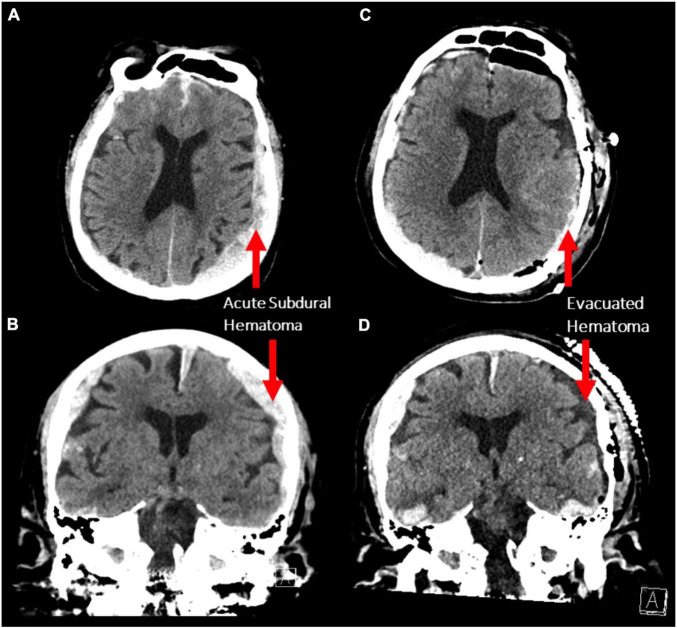
**(A,B)** Axial and coronal non-contrast CT scans demonstrating bilateral acute subdural hematoma with a larger mass effect on the left side (maximum thickness 1.5 cm). **(C,D)** The same scan sequences after decompressive craniotomy demonstrating evacuation of the left subdural hematoma.

**FIGURE 2:**
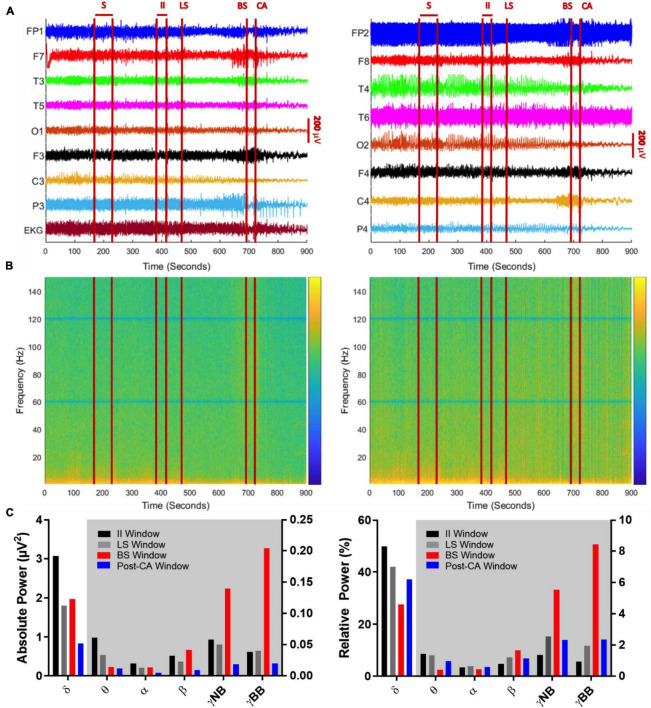
**(A)** Global EEG output from the 10–20 system with concurrent EKG signal over a 900 s period encompassing a seizure (S), suppression of left cerebral hemisphere activity (LS), suppression of bilateral cerebral hemisphere activity (BS), and cardiac arrest (CA). Left panel indicates left brain hemisphere, right panel indicates right brain hemisphere. **(A)** 240 s (sec) after termination of seizure activity (time window: 170–230 s), neuronal activity ceases on the left side (at 470 s), before both hemispheric suppression (694 s) and cardiac arrest occurs (at 720 s). Between BS and CA, there is an acute decline in EEG amplitude captured by multiple leads (FP1, F7, P3, FP2, T4). After CA, there is a global decline in EEG amplitude. **(B)** Spectrograms of absolute (left) and relative (right) power of global EEG (16 channels) over the 900-s recording. The z-axis of the spectrograms utilizes a log scale heat map with blue indicating low power and yellow indicating high power. The notch filter applied at 60 Hz and its super harmonics are visible as solid horizontal blue lines. Absolute and relative gamma band power is more dominant in the period between BS and CA, while low frequency activity (< 25 Hz) declines across the recorded time. **(C)** Mean absolute (left) and relative (right) power of sequential frequency bands taken in 30-s epochs centered over the midpoints of EEG activity between seizure activity and left cerebral suppression (II window: 385–415 s), suppression in left and bilateral hemispheres (LS window: 510–540 s), between bilateral suppression and cardiac arrest (BS window: 690–720 s) and finally, cardiac arrest and the end of the recording (post-CA window: 810–840 s). The white background indicates bars belonging to the left y-axis and the gray background indicates bars belonging to the right y-axis.

An 87-year-old male presented to the emergency department after a fall. Initially, his Glasgow Coma Scale (GCS) was 15, however, he rapidly deteriorated to a GCS of 10 (E3V2M5) with anisocoria (left pupil:4 mm, right pupil: 2 mm) and bilateral reaction to light. Corneal and gag reflexes were preserved. The CT scan demonstrated bilateral acute subdural hematomas (SDH), with a larger mass effect on the left side (maximal diameter: 1.5 cm; Figures 1A,B) and midline-shift. Due to the radiographic finding and the patient’s precipitous decline in neurologic status, a left decompressive craniotomy was performed to evacuate the hematoma. Post-operatively, the patient was stable for two days during his stay in the intensive care unit, before he deteriorated with flexor posturing, worsened right hemiparesis and intermittent myoclonic jerks in his lower extremities. A CT scan was completed, demonstrating successful evacuation of the left SDH and a stable right-sided hematoma (Figures 1C,D). After neurological consultation, the patient received phenytoin and levetiracetam and an electroencephalography (EEG) was obtained, which showed non-convulsive status epilepticus in the left hemisphere. There were at least 12 identified electrographic seizures, after which a burst suppression pattern spontaneously developed over the left hemisphere (Figure 2A). Shortly thereafter, electrographic activity over both hemispheres demonstrated a burst suppression pattern, which was followed by development of ventricular tachycardia with apneustic respirations and clinical cardiorespiratory arrest. After discussion with the patient’s family and in consideration of the “Do-Not-Resiscitate (DNR)” status of the patient, no further treatment was administered and the patient passed away.

## Materials and Methods

### Electroencephalography Recording

In the peri-arrest period, EEG signals were recorded at 512 Hz using the standard 10-20 system of electrode placement. The reference electrode is placed in the frontal vertex region and bilateral auricular electrodes are used for the referential montage. The pertinent 900 s of unprocessed EEG data in the peri-arrest period from 8 left-sided leads and 8 analogous right-sided leads, as well as the patient’s EKG tracing is shown in Figure 2A. Electroencephalography interpretation is based on the American Clinical Neurophysiology Society Standardized Critical Care EEG Terminology (Hirsch et al., 2021). Electrographic seizures were documented if epileptiform discharges averaged > 2.5 Hz for > 10 s. Suppression of activity over the left and right hemispheres is defined as the conversion of EEG background into a burst-suppression or suppression pattern without medical intervention or return to prior baseline. Due to the acute onset, no prior EEG recording was captured to represent normal activity. Cardiac arrest is defined as the abrupt loss of heart function measured by the inability to obtain pulse activity in the ECG.

### Spectral Power Analysis

A notch filter is applied to remove the 60 Hz line noise and its super harmonics. EEG power spectrum is calculated by applying a fast Fourier transform to create a spectrogram using the spectrogram.m tool (Matlab Signal Processing Toolbox; Mathworks Inc., Natick, United States) with a 2-s epoch size and 1 s overlapping each epoch (Figure 2B). Epochs are windowed using a Hamming window. The absolute power is expressed in log scale and the relative power is calculated over each epoch by dividing the absolute power captured in a single frequency band by the total absolute power summed over all frequency bands. For spectral power analysis, frequency bands are defined as delta (0.5–5 Hz), theta (5–10 Hz), alpha (10–15 Hz), beta (15–25 Hz), narrow-band gamma (30–60 Hz) and broad-band gamma (80–150 Hz). The absolute and relative mean spectral power of global EEG is calculated over 4 windows of interest, each 30 s of duration: (i) the interictal interval (385–415 s) is defined as the time window around the midpoint between the end of the seizure interval (S) and the suppression of left-sided activity, (ii) the window for left hemispheric suppression (LS) (510–540 sec) is determined as a 30 sec interval occurring after suppressed activity in the left hemisphere, (iii) the bilateral suppression (BS) of activity window (690–720 sec) is identified as the 30 sec temporal interval between suppression of bilateral responses (BS) and cardiac arrest (CA) and finally, (iv) the post-cardiac arrest window (810–840 s) is characterized as the 30 sec interval between cardiac arrest to the end of the recording.

### Cross-Frequency Coupling and Coherence Analysis

Phase-amplitude coupling is measured by the mean vector length modulation index (Canolty et al., 2006), which quantifies how much the amplitude of a signal at a frequency band is modulated by the phase of another band. For this analysis the instantaneous phase and amplitude of band-filtered EEG signals are determined by the Hilbert transform (Le Van Quyen et al., 2001; Aru et al., 2015). The instantaneous phase and amplitude are used to build a complex valued signal by multiplying the instantaneous amplitude by the complex exponential of the instantaneous phase. The mean vector length of this complex signal indicates whether the instantaneous amplitude is consistently higher or lower for specific values of the phase. The modulation index (MI) is finally obtained by comparing the mean vector length of the real signals with the distribution of vector lengths of shuffled surrogate data that selectively destroys the phase-amplitude correlations and it is measured in standard deviations of the surrogate distribution.

Coherence between pairs of electrodes belonging to different regions was measured by their magnitude-squared coherence. This measure calculates the normalized magnitude-squared of the cross-spectrum of two signals at each frequency. The estimation of coherence was obtained using the tool mscohere.m (Matlab Signal Processing Toolbox; Mathworks Inc., Natick, United States). The tool was applied to pairs of EEG signals of distinct electrodes segmented in 2-s epochs with 1 s overlapping each epoch as previously shown (Borjigin et al., 2013). Coherence for each frequency band was obtained by averaging over the range of frequency bins of each band. Global values of coherence for the windows of interest were obtained by averaging the pair-wise coherences across pairs of distinct electrodes.

## Results

To investigate oscillatory changes during NDE, we analyzed neuronal oscillations from EEG recordings obtained during the transition period to death. We analyzed four time windows of interest: (1.) The interictal interval (II) window captures activity from 385 to 415 s after the clinical seizure. (2.) The left suppression (LS) window targets global spectral power from 510 to 540 s at the midpoint between suppression of left and bilateral hemispheric activity. (3.) The bilateral suppression (BS) window spans an interval between suppression of bilateral hemispheric activity and clinical cardiac arrest from 690 to 720 s. (4.) The final window includes the post-cardiac arrest (post-CA) period from 810 to 840 s at the midpoint between cardiac arrest and the end of the EEG recording. A timeline with these windows is shown in Figures 2A,B.

### Changes in Power Spectrum of High-Frequency Gamma Bands

Absolute (Figure 2B, left panel) and relative spectral power (Figure 2B, right panel) of low frequency activity less than 25 Hz declines over the course of the EEG recording. When bilateral hemispheric activity ceases, there is a temporary increase in absolute narrow- and broad-band gamma power, which declines after clinical cardiac arrest. Absolute and relative spectral power were quantified for delta (0.5–5 Hz), theta (5–10 Hz), alpha (10–15 Hz), beta (15–25 Hz), narrow-band gamma (30–60 Hz) and broad-band gamma (80–150 Hz) over the 4 windows of interest (Figure 2C). A surge of gamma-band power is seen after suppression of bilateral hemispheric activity (Figure 2C, red bars for narrow and broad gamma bands in left panel; II absolute γNB: 0.06μV^2^, II absolute γBB: 0.04μV^2^, BS absolute γNB: 0.14μV^2^, BS absolute γBB: 0.2μV^2^). This increment is also identified for the relative power of narrow and broad-band gamma, the percentage of power in such bands compared to the total power summed across all bands considered (Figure 2C, red bars for narrow and broad gamma bands in left and right panel; II relative γNB: 1.37%, II relative γBB: 0.94%, BS relative γNB: 5.56%, BS relative γBB: 8.44%). In other words, both, the gamma power and its percentage over the total spectral power increase after bilateral hemispheric activity suppression. Post cardiac arrest, absolute power of gamma activity is decreased compared to all previous time windows (Figure 2C, blue bars for narrow and broad gamma bands in left panel; post-CA absolute γNB: 0.02μV^2^, post-CA absolute γBB: 0.02μV^2^). On the contrary, the relative power spectrum reveals an increase of relative gamma power activity when compared to the interictal interval (Figure 2C, black vs blue bars for narrow and broad gamma bands in right panel; II relative γNB: 1.37%, II relative γBB: 0.94%, post-CA relative γNB: 2.33%, Post-CA relative γBB: 2.35%).

### Modulation of Delta, Theta, Alpha and Beta Bands

In addition, a prominent decline is seen in the delta-band from the interictal interval period (3.07 μV^2^) to LS (1.80 μV^2^) and a further decay in the post-CA period (0.84 μV^2^) (Figure 2C, left, gray and blue bars in the delta-band). Relative power of delta activity declines from 42.0% in LS to 27.6% in BS to compensate for the increase in narrow-band gamma and broad-band gamma activity after BS with an eventual rebound elevation in relative delta (37.3%) in post-CA (Figure 2C right panel, gray, red and blue bars in the delta band). Absolute theta activity is decreased after left and bilateral hemispheric suppression and remains stable in the post-CA period (Figure 2C left panel; theta II: 0.0616 μV^2^, LS: 0.0339 μV^2^, BS: 0.0143 μV^2^, post-CA: 0.0121 μV^2^), while the sole change in the relative theta band is seen after bilateral activity suppression (Figure 2C, right panel, red bar; theta II: 1.44%, LS: 1.36%, BS: 0.44%, post-CA: 0.98%). In the alpha and beta bands, the most prominent alteration is a decline in absolute power after cardiac arrest (Figure 2C, left panel, alpha II: 0.0201 μV^2^, LS: 0.0135 μV^2^, BS: 0.0140 μV^2^, post-CA: 0.0053 μV^2^; beta II: 0.0326 μV^2^, LS: 0.0229 μV^2^, BS: 0.0415 μV^2^, post-CA: 0.0093 μV^2^). A summary of all absolute and relative spectral power values across all investigated bands is provided in Table 1.

**TABLE 1:** Raw values for absolute (μV^2^) and relative (%) power of sequential frequency bands: delta (0.5–5 Hz), theta (5–10 Hz), alpha (10–15 Hz), beta (15–25 Hz), narrow-band gamma (γNB, 30–60 Hz) and broad-band gamma (γBB 80–150 Hz).

Absolute Power (μ V^2^)	δ	θ	α	β	γ NB	γ BB
II Window	3.0659	0.0615	0.0200	0.0326	0.0583	0.0382
LS Window	1.7987	0.0339	0.0134	0.0228	0.0506	0.0402
II Window	1.9736	0.0142	0.0140	0.0414	0.1399	0.2041
Post-CA Window	0.8381	0.0121	0.0053	0.0093	0.0189	0.0198

**Relative Power (%)**	δ	θ	α	β	**γ NB**	**γ BB**

II Window	49.86	1.43	0.58	0.78	1.36	0.93
LS Window	41.96	1.35	0.65	1.22	2.55	1.95
BS Window	27.65	0.44	0.46	1.65	5.55	8.43
Post-CA Window	37.32	0.98	0.61	1.14	2.33	2.34

*Each value is the average of 30-s epochs centered over the midpoints of the following intervals: Interictal interval (II) window: Between Seizure (S) and Left Hemispheric Suppression (LS), LS window: Between LS and Bilateral Hemispheric Suppression (BS), BS window: Between BS and cardiac arrest (CA) and post-CA window: From CA to the end of the recording.*

### Cross-Frequency Coupling and Coherence

**FIGURE 3:**
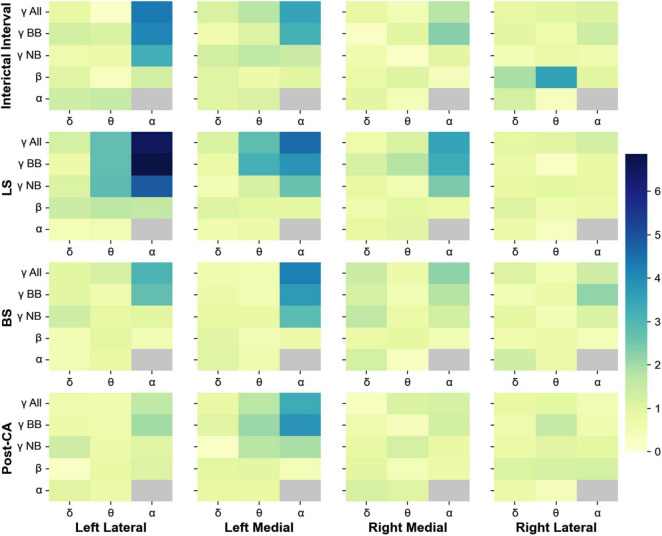
Phase-amplitude cross-frequency co-modulogram computed during the interictal interval (II), ceased left hemisphere activity (LS), suppression of bilateral activity (BS) and post cardiac arrest (post-CA). Column 1 represents the lateral left brain hemisphere, column 2 shows the left medial electrodes. The 3^rd^ and 4^th^ columns depict the electrodes representing the right medial and right lateral hemisphere, respectively. For each pair of frequency bands, the color codes for the phase-amplitude modulation index. X-axis indicates the band selected for phase extraction and y-axis indicates the band selected for amplitude extraction. During the interictal interval window, the phase of the alpha band activity modulates the power at gamma and broadband activity of the lateral electrodes. During suppression of left hemispheric activity, the phase of theta and alpha bands modulate the amplitude at the gamma band for medial and lateral electrodes in the left hemisphere. Finally, during bilateral activity ceases and post-CA, the alpha band phase continues modulating gamma and broadband power in left medial electrodes.

Phase-amplitude cross-frequency coupling has been used to determine the strength with which the phase of one frequency band modulates the amplitude of another (Canolty et al., 2006). This type of coupling is thought to reflect the strength of interactions between neuronal processes occurring at different bands (Canolty et al., 2006; Jensen and Colgin, 2007; Aru et al., 2015). In addition to the changes in spectral power, we also analyzed the phase-amplitude cross-frequency coupling between for all 4 windows of interest (II, LS, BS and post-CA). Figure 3 shows the indices for the modulation of the amplitudes of higher frequency bands (alpha, beta, and gamma) by the phases of low-frequency bands (delta, theta, and alpha). Gamma bands in the left hemisphere modulate their power according to the phases of alpha and theta rhythms across all windows including the interval following cardiac arrest. The strongest coupling is observed for the modulation of the amplitude of narrow-band gamma (MI = 4.8, p_*value*_ = 7.9 × 10^–7^) and broadband gamma (MI = 6.9, p_*value*_ < 1 × 10^–9^) by the phase of alpha band activity during the suppression of left hemispheric activity (LS). A summary of all modulation indices for phase-amplitude coupling across all investigated bands is provided in Table 2.

**TABLE 2:** Modulation indices for phase-amplitude coupling within electrodes across the 4 windows of interest [Interictal interval (II) window: Between Seizure (S) and Left Hemispheric Suppression (LS), LS window: Between LS and Bilateral Hemispheric Suppression (BS), BS window: Between BS and cardiac arrest (CA) and post-CA window: From CA to the end of the recording].

Left lateral electrodes			
	δ	**θ**	α
**Interictal interval Window**			
γ All	0,865	0,191	4.34
γ BB	1.306	1.162	4.116
γ NB	0,675	0,741	3.263
β	1.035	0,365	1.29
α	1.381	1.51	
**LS Window**			
γ All	1.285	2.755	6.502
γ BB	0,723	2.784	6.866
γ NB	1.132	2.841	4.831
β	1.431	1.674	1.607
α	0,465	0.57	
**BS Window**			
γ All	0,996	1.215	3.06
γ BB	0,981	0,591	2.759
γ NB	1.373	0,876	0,992
β	0.51	0,955	0,524
α	0,489	0,823	
**Post-CA Window**			
γ All	0,682	0,628	1.615
γ BB	0,739	0,671	1.979
γ NB	1.365	0.7	1.062
β	0,259	0,811	1.119
α	1.04	0,744	

**Left medial electrodes**			
	δ	**θ**	α

**Interictal interval Window**			
γ All	1.18	1.748	3.526
γ BB	0	1.111	3.186
γ NB	1.292	1.622	1.429
β	1.049	0.75	1.008
α	1.064	1.206	
**LS Window**			
γ All	1.159	2.803	4.567
γ BB	0,799	3.2	3.811
γ NB	0.52	1.214	2.649
β	1.08	0,959	0,938
α	0,637	0,734	
**BS Window**			
γ All	0,594	0,585	4.239
γ BB	0,706	0,538	3.71
γ NB	0,863	0,852	2.843
β	1.02	0,569	0,713
α	1.059	0,623	

**Right medial electrodes**			
	δ	**θ**	α

**Post-CA Window**			
γ All	0,895	1.741	3.34
γ BB	1.041	2.081	3.832
γ NB	0,225	1.748	1.922
β	0.96	0,994	0,479
α	0,903	0,828	
**Interictal interval Window**			
γ All	0.89	0,541	1.757
γ BB	0,231	1.003	2.294
γ NB	0,611	0,204	0,995
β	0,804	1.078	0,429
α	0,999	0,479	
**LS Window**			
γ All	0,687	1.158	3.525
γ BB	1.209	1.786	3.329
γ NB	0,867	0,583	2.427
β	0,628	0,943	0,804
α	0,877	1.022	
**BS Window**			
γ All	1.423	0,701	2.212
γ BB	1.231	0,553	1.775
γ NB	1.661	0,797	1.301
β	0.77	0,924	0,528
α	1.268	0,268	
**Post-CA Window**			
γ All	0,308	1.156	1.218
γ BB	0,654	0,381	1.336
γ NB	0.86	1.25	0,826
β	1.012	0,468	0,744
α	1.223	1.056	

**Right lateral electrodes**			
	δ	**θ**	α

**Interictal interval Window**			
γ All	0,866	0,992	1.158
γ BB	0,972	0,693	1.376
γ NB	0,521	0,783	0,636
β	1.908	3.565	0,997
α	1.261	358	
**LS Window**			
γ All	0,931	1.024	1.308
γ BB	0,783	0,265	0,834
γ NB	0,823	0,954	0,866
β	1.084	0,623	0,776
α	0,713	0,353	
**BS Window**			
γ All	1.098	0.57	1.354
γ BB	0,562	0,726	2.198
γ NB	0,929	0,535	1.154
β	0,683	0,711	0,462
α	1.349	0,732	

**Right medial electrodes**			
	δ	**θ**	α

**Post-CA Window**			
γ All	0,863	0,955	0,582
γ BB	0,662	1.562	0,631
γ NB	0,758	1.04	0,962
β	1.131	1.215	1.287
α	0,737	0,434	

*The table shows the modulation index for the coupling between the phase of slower frequency bands [delta (0.5–5 Hz), theta (5–10 Hz), alpha (10–15 Hz)] to the amplitude of alpha, beta (15–25 Hz), narrow-band gamma (γNB, 30–60 Hz), broad-band gamma (γBB 80–150 Hz), and the whole gamma band (γAll, 30–150 Hz).*

**FIGURE 4:**
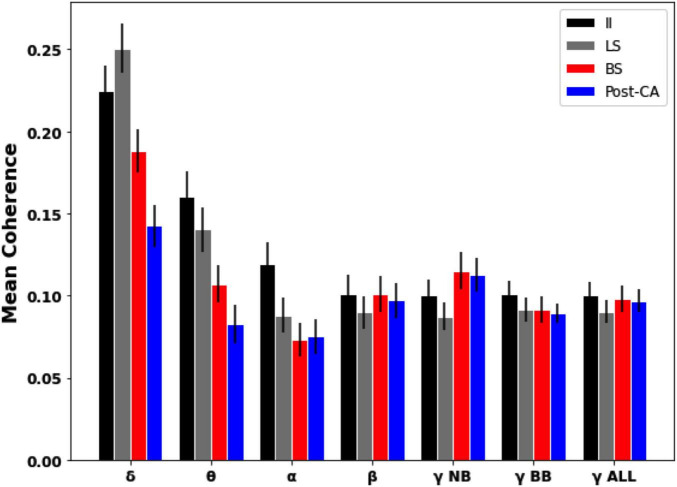
Mean pairwise coherence of EEG dynamics across different intervals and frequency bands. Mean was computed over the pairwise coherences between electrodes in different regions. Error bars indicate the standard error of the mean. Slower bands (delta, theta, and alpha) show a decrease in coherence following cardiac arrest (Post-CA) compared to interictal (II) and suppression of left hemispheric activity (LS). Faster frequency bands show an almost unchanged level of mean coherence with a small increase in narrow-gamma coherence following bilateral suppression (BS) and cardiac arrest (Post-CA) compared to earlier epochs.

Coherence analysis between different electrodes was also used to estimate frequency-resolved dependencies across EEG signals. Figure 4 shows the magnitude-squared coherence averaged across pairs of distinct electrodes for each of the four time windows of interest. For our data, it is observed that for slower frequency bands (delta, theta, and alpha) the global coherence following cardiac arrest (Post-CA) is diminished compared to its values for interictal (II) and left hemispheric suppression (LS) intervals. For faster frequency bands (beta and gamma), the coherence is observed to remain almost unchanged across the different intervals with the exception of a small increase for narrow-band gamma during bilateral suppression (BS) and following cardiac arrest (Post-CA) when compared to earlier stages.

## Discussion

We report continuous EEG recording from a human brain across the transition period to death. The spectral analysis revealed a surge in absolute gamma power after suppression of neuronal activity in both hemispheres, followed by a marked decrease after cardiac arrest. In relative terms, the percentage gamma power over the total signal power is also increased after bilateral suppression together with a reduction of theta rhythms. After cardiac arrest, the relative amount of gamma power increased compared to the interictal interval, while a reduction of delta, beta, alpha and absolute gamma waves was identified. Cross-frequency coupling revealed strong modulation of the low- and broad gamma power by the alpha band. Inter-regional coherence analysis showed that after cardiac arrest, a reduction in global coherence occurred for the slower frequency bands while the coherence for faster bands was unchanged or slightly enhanced for narrow-band gamma. Together, these findings suggest that an intricate interplay between low- and high-frequency bands takes place after gradual cessation of cerebral activity and lasts into the period when cerebral blood flow is ceased (post cardiac arrest).

The findings we report here are similar to the alterations in neuronal activity that have been observed in rodents, where an increase of low gamma band frequencies was observed 10–30 s after cardiac arrest (Borjigin et al., 2013). Our data reveals enhanced relative gamma power compared to other bands along with a decrease in theta. An interesting difference between the two studies can be observed when comparing phase-amplitude coupling (cross-frequency coupling): Post cardiac arrest, delta, theta, and alpha modulate low gamma activity in the rodent (Borjigin et al., 2013), whereas in the human brain, such modulation occurs in all gamma bands and is mostly mediated by alpha waves, to a lesser degree by theta rhythms. The alpha band is thought to critically interfere in cognitive processes by inhibiting networks that are irrelevant or disruptive (Klimesch, 2012). Given that cross-coupling between alpha and gamma activity is involved in cognitive processes and memory recall in healthy subjects, it is intriguing to speculate that such activity could support a last “recall of life” that may take place in the near-death state. Unlike previous reports, our study is the first to use full EEG placement, which allows a more complete neurophysiological analysis in a larger dimension. Further, the data was obtained from an acutely deteriorating patient. Previous human reports were limited to frontal cortex EEG signals that were analyzed by neuromonitoring devices, which may have captured artifacts and the focus was set on critically ill patients in chronic settings (Chawla et al., 2009, 2017). In line with our findings, electrical surges were also reported in these studies after cessation of blood circulation.

In considering how these findings might be generalized to understand the typical patterns of brain activity during death, there are several caveats that must be considered. First, an important consideration is the patient’s post-traumatic brain that suffered hemorrhage, swelling and seizures. Traumatic brain injury (TBI) and white matter damage can influence rhythmic brain activity (Sanchez et al., 2019). Post-injury, network activity is acutely decreased, which initiates upregulatory mechanisms to enhance cortical excitability (Avramescu and Timofeev, 2008). When deprived of oxygen, cells go through a brief phase of enhanced excitability, and the brain generates the activity patterns that are dictated by its connectome (Fields et al., 2015). These changes in network excitability can increase synchronization after partial deafferentation or trauma-induced epilepsy (Avramescu and Timofeev, 2008; Timofeev et al., 2013). It has been argued that the default emergent activity patterns of the cortical network are highly synchronous slow-waves, arising after the cortex is physically or functionally disconnected from external stimulation, e.g., during deep sleep, anesthesia or in *in vitro* slice preparations (Sanchez-Vives and McCormick, 2000; Steriade et al., 2001; Chauvette et al., 2011). Second, anesthesia-induced loss of consciousness can alter neuronal oscillations, including alpha waves (Ching et al., 2010) and an increase phase synchronization of gamma oscillations (Murphy et al., 2011). Third, dissociative drugs (Lee et al., 2017; Li et al., 2019) and psychosis are linked to a surge in gamma synchronization as observed in schizophrenia (Hirano et al., 2015), opening the possibility that dissociative events and drugs can cause an increase in gamma activity. Fourth, the patient had been placed on significant doses of anticonvulsant medication, which could directly affect the neuronal network activity (Lanzone et al., 2020). Fifth, asphyxia and hypercapnia can enhance cortical connectivity. Especially the pre-arrest surge in gamma synchronization observed in rodents (Li et al., 2015; Zhang et al., 2019) and in humans as seen in this study could be caused by hypercapnia and resulting acidosis, which may stimulate gap-junction activity, that is critical for gamma oscillations. Sixth, no normal activity was recorded with the EEG that can serve as a true baseline for comparison. Finally, while stereotyped neuronal activity patterns are conserved during daily behavioral tasks, it is not researched whether a similar evolutionary constraint demanding a proscribed process is present during the transition phase to death.

Despite these caveats, the overall similarity in oscillatory changes between the highly controlled experimental rodent study and the present work suggests that the brain may pass through a series of stereotyped activity patterns during death. It may ultimately be difficult to assess this in a physiological environment, since gathering such data from “healthy-subjects” is impossible by definition. We do not anticipate death in healthy subjects and therefore could not obtain uninterrupted recordings in the near-death phase in anything other than from circumstances involving pathological conditions in acute care hospital settings.

## Data Availability Statement

The original contributions presented in the study are included in the article/Supplementary Material, further inquiries can be directed to the corresponding author.

## Ethics Statement

Ethical review and approval was not required for the study on human participants in accordance with the local legislation and institutional requirements. The patients/participants provided their written informed consent to participate in this study.

## Author Contributions

RV, MR, RL, AL, JN, and AZ wrote the manuscript. RV, MR, TK, FM-A, and AZ analyzed data. CS, KY, MS, MF, and CH provided data and revised the manuscript. All authors contributed to the article and approved the submitted version.

## Conflict of Interest

The authors declare that the research was conducted in the absence of any commercial or financial relationships that could be construed as a potential conflict of interest.

## Publisher’s Note

All claims expressed in this article are solely those of the authors and do not necessarily represent those of their affiliated organizations, or those of the publisher, the editors and the reviewers. Any product that may be evaluated in this article, or claim that may be made by its manufacturer, is not guaranteed or endorsed by the publisher.
