# Supplementary material for: Enhanced Interplay of Neuronal Coherence and Coupling in the Dying Human Brain
Source: Front Aging Neurosci. 2022 Feb 22;14:813531. doi: 10.3389/fnagi.2022.813531 (PMC8902637; doi:10.3389/fnagi.2022.813531)
Supplement: Supplementary file 1 [file Data_Sheet_1.pdf]

Supplementary Figure 1. EEG recordings from the standard 10-20 system (Natus NeuroWorks 8, Natus Medical Inc., Middleton, WI). (A) This is a representative sample of an EEG performed on the same patient approximately 7 hours prior to death. Left-sided continuous rhythmic activity with delta waves and superimposed faster rhythms is observed. Due to the acuity of the patient's illness and lack of prior neurologic pathology, this is the only baseline study available for comparison. (B) The patient experienced a total of 12 seizures prior to death, this is a representative EEG montage of the 12th seizure which occurs in the 900-second representative EEG window between 170-230 seconds. (C) Immediately prior to the final seizure, periodic 1 Hz sharp waves are seen at lead T3. (D) After the final seizure a modified burst suppression pattern is detected. The periodic artifact visible is due to respiration.

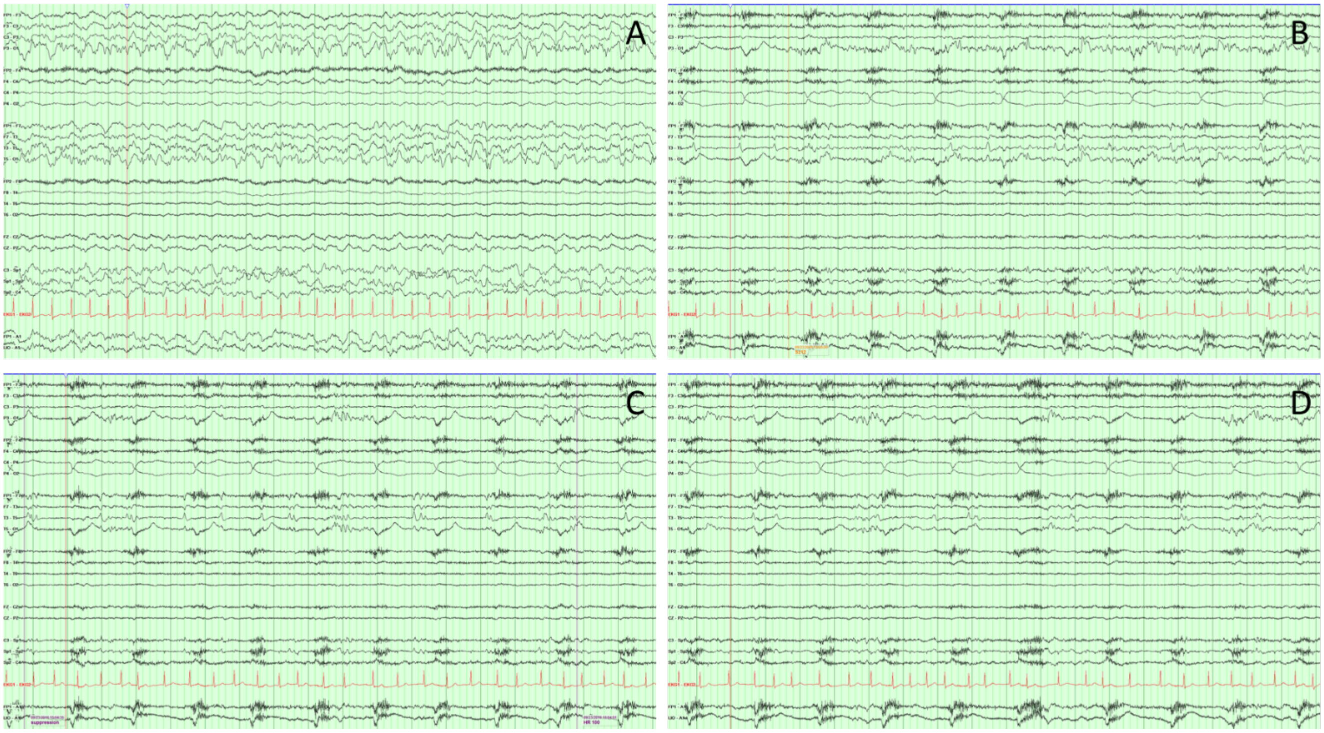

Supplementary Figure 2. Global EEG output from the 10-20 system of electrodes over the first 5 seconds (415-420 seconds) of the inter-ictal period denoted in Figure 2 from all left sided leads (A) and right-sided leads (B). EEG output over the first 5 seconds (810-815 seconds) of the Post-CA period denoted in Figure 2 from all left-sided leads (C) and all right-sided leads (D). Amplitude of raw EEG in the Post-CA period is globally lower than during the analogous 5 seconds of the inter-ictal period.

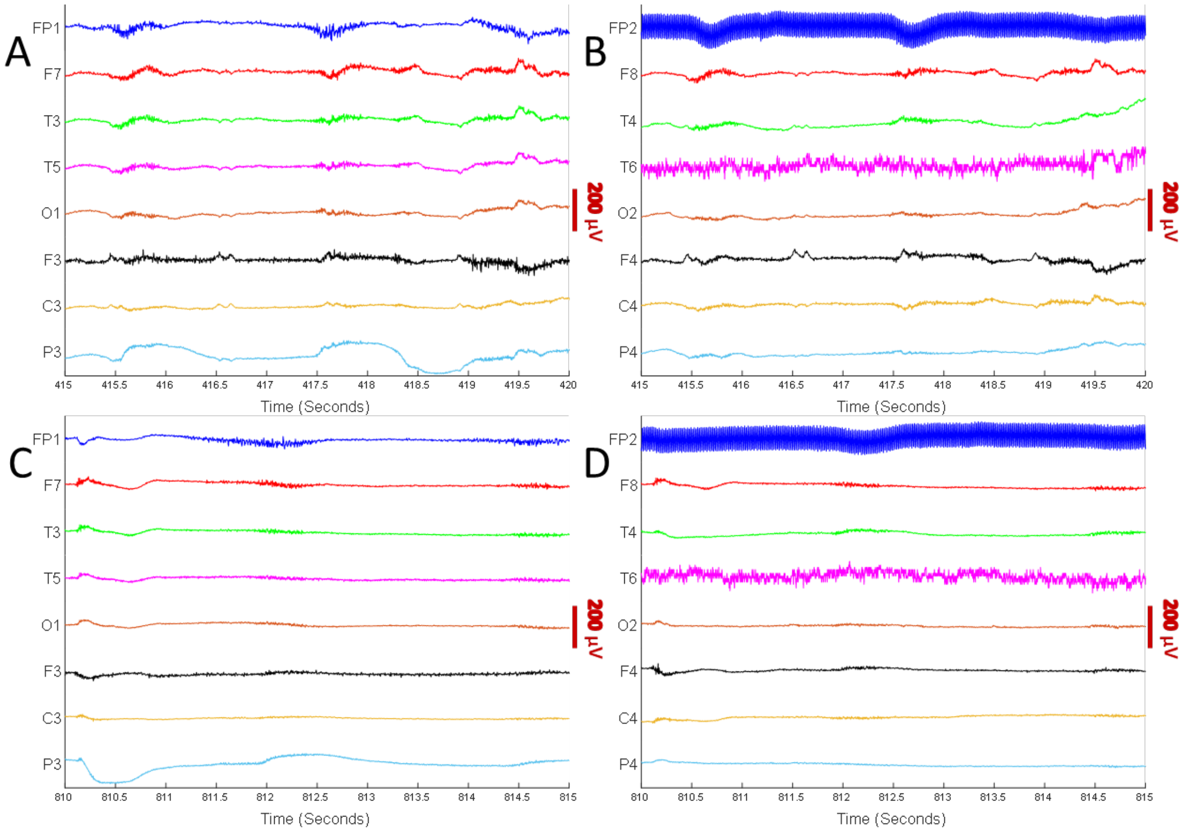

Supplementary Table 1

Absolute Power (μV^2)	δ	θ	α	β	γNB	γBB
**II Window**	3.0659	0.0615	0.0200	0.0326	0.0583	0.0382
**LS Window**	1.7987	0.0339	0.0134	0.0228	0.0506	0.0402

**II Window**	1.9736	0.0142	0.0140	0.0414	0.1399	0.2041
**Post-CA Window**	0.8381	0.0121	0.0053	0.0093	0.0189	0.0198

**Relative Power (%)**	**δ**	**θ**	**α**	**β**	**γNB**	**γBB**
**II Window**	49.86	1.43	0.58	0.78	1.36	0.93
**LS Window**	41.96	1.35	0.65	1.22	2.55	1.95
**BS Window**	27.65	0.44	0.46	1.65	5.55	8.43
**Post-CA Window**	37.32	0.98	0.61	1.14	2.33	2.34

Supplementary Table 1. Raw values for absolute (μV^2) and relative (%) power of sequential frequency bands: delta (0.5-5 Hz), theta (5-10 Hz), alpha (10-15 Hz), beta (15-25 Hz), narrow-band gamma (γNB, 30-60 Hz) and broad-band gamma (γBB 80-150 Hz). Each value is the average of 30-second epochs centered over the midpoints of the following intervals: Interictal interval (II) window: Between Seizure (S) and Left Hemispheric Suppression (LS), LS window: Between LS and Bilateral Hemispheric Suppression (BS), BS window: Between BS and cardiac arrest (CA) and post-CA window: From CA to the end of the recording.

Supplementary Table 2

**Left lateral electrodes**			
**Interictal interval Window**			
	δ	θ	α
γ All	0,865	0,191	4.34
γ BB	1.306	1.162	4.116
γ NB	0,675	0,741	3.263
β	1.035	0,365	1.29
α	1.381	1.51	
**LS Window**			
γ All	1.285	2.755	6.502
γ BB	0,723	2.784	6.866
γ NB	1.132	2.841	4.831
β	1.431	1.674	1.607
α	0,465	0.57	
**BS Window**			
γ All	0,996	1.215	3.06
γ BB	0,981	0,591	2.759
γ NB	1.373	0,876	0,992

β	0.51	0,955	0,524
α	0,489	0,823	
**Post-CA Window**			
γ All	0,682	0,628	1.615
γ BB	0,739	0,671	1.979
γ NB	1.365	0.7	1.062
β	0,259	0,811	1.119
α	1.04	0,744	

**Left medial electrodes**			
**Interictal interval Window**			
	δ	θ	α
γ All	1.18	1.748	3.526
γ BB	0	1.111	3.186
γ NB	1.292	1.622	1.429
β	1.049	0.75	1.008
α	1.064	1.206	
**LS Window**			

γ All	1.159	2.803	4.567
γ BB	0,799	3.2	3.811
γ NB	0.52	1.214	2.649
β	1.08	0,959	0,938
α	0,637	0,734	
**BS Window**			
γ All	0,594	0,585	4.239
γ BB	0,706	0,538	3.71
γ NB	0,863	0,852	2.843
β	1.02	0,569	0,713
α	1.059	0,623	
**Post-CA Window**			
γ All	0,895	1.741	3.34
γ BB	1.041	2.081	3.832
γ NB	0,225	1.748	1.922
β	0.96	0,994	0,479
α	0,903	0,828	

**Right medial electrodes**			
**Interictal interval Window**			
	δ	θ	α
γ All	0.89	0,541	1.757
γ BB	0,231	1.003	2.294
γ NB	0,611	0,204	0,995
β	0,804	1.078	0,429
α	0,999	0,479	
**LS Window**			
γ All	0,687	1.158	3.525
γ BB	1.209	1.786	3.329
γ NB	0,867	0,583	2.427
β	0,628	0,943	0,804
α	0,877	1.022	
**BS Window**			
γ All	1.423	0,701	2.212
γ BB	1.231	0,553	1.775
γ NB	1.661	0,797	1.301
β	0.77	0,924	0,528

α	1.268	0,268	
**Post-CA Window**			
γ All	0,308	1.156	1.218
γ BB	0,654	0,381	1.336
γ NB	0.86	1.25	0,826
β	1.012	0,468	0,744
α	1.223	1.056	

**Right lateral electrodes**			
**Interictal interval Window**			
	δ	θ	α
γ All	0,866	0,992	1.158
γ BB	0,972	0,693	1.376
γ NB	0,521	0,783	0,636
β	1.908	3.565	0,997
α	1.261	358	
**LS Window**			
γ All	0,931	1.024	1.308

γ BB	0,783	0,265	0,834
γ NB	0,823	0,954	0,866
β	1.084	0,623	0,776
α	0,713	0,353	
**BS Window**			
γ All	1.098	0.57	1.354
γ BB	0,562	0,726	2.198
γ NB	0,929	0,535	1.154
β	0,683	0,711	0,462
α	1.349	0,732	
**Post-CA Window**			
γ All	0,863	0,955	0,582
γ BB	0,662	1.562	0,631
γ NB	0,758	1.04	0,962
β	1.131	1.215	1.287
α	0,737	0,434	

Supplementary Table 2. Modulation indices for phase-amplitude coupling within electrodes across the 4 windows of interest (Interictal interval (II) window: Between

Seizure (S) and Left Hemispheric Suppression (LS), LS window: Between LS and Bilateral Hemispheric Suppression (BS), BS window: Between BS and cardiac arrest (CA) and post-CA window: From CA to the end of the recording.). The table shows the modulation index for the coupling between the phase of slower frequency bands (delta (0.5-5 Hz), theta (5-10 Hz), alpha (10-15 Hz)) to the amplitude of alpha, beta (15-25 Hz), narrow-band gamma (γNB, 30-60 Hz), broad-band gamma (γBB 80-150 Hz), and the whole gamma band (γAll, 30-150 Hz).
